# Cracking Brain Diseases from Gut Microbes-Mediated Metabolites for Precise Treatment

**DOI:** 10.7150/ijbs.85259

**Published:** 2023-06-04

**Authors:** Ying Gong, Anmei Chen, Guohui Zhang, Qing Shen, Liang Zou, Jiahong Li, Yang-Bao Miao, Weixin Liu

**Affiliations:** 1 Department of Haematology, Sichuan Academy of Medical Sciences & Sichuan Provincial People's Hospital, School of Medicine of University of Electronic Science and Technology of China, No. 32, West Section 2, First Ring Road, Qingyang District, Chengdu 610000, China; 2 Key Laboratory of reproductive medicine, Sichuan Provincial maternity and Child Health Care Hospital, Chengdu 610000, China.; 3 School of Life Science and Engineering, Southwest Jiaotong University, Chengdu 610041, China; 4 School of Food and Biological Engineering, Chengdu University, Chengdu 610106, Sichuan, China.

**Keywords:** Gut Microbes, Metabolites, Gut to Brain, Vagus Nerve, Immune System

## Abstract

The gut-brain axis has been a subject of significant interest in recent years. Understanding the link between the gut and brain axis is crucial for the treatment of disorders. Here, the intricate components and unique relationship between gut microbiota-derived metabolites and the brain are explained in detail. Additionally, the association between gut microbiota-derived metabolites and the integrity of the blood-brain barrier and brain health is emphasized. Meanwhile, gut microbiota-derived metabolites with their recent applications, challenges and opportunities their pathways on different disease treatment are focus discussed. The prospective strategy of gut microbiota-derived metabolites potential applies to the brain disease treatments, such as Parkinson's disease and Alzheimer's disease, is proposed. This review provides a broad perspective on gut microbiota-derived metabolites characteristics facilitate understand the connection between gut and brain and pave the way for the development of a new medication delivery system for gut microbiota-derived metabolites.

## Introduction

The brain and gut microbiota have a close relationship, with gut microbes significantly influencing the brain. Additionally, the gut and the brain share many similarities and characteristics, including the presence of numerous neurons and glial cells that provide support and nourishment to neurons. Within the human body, the gut is responsible for producing more than forty neurotransmitters, including fifty percent of the body's dopamine and ninety-five percent of its serotonin 1. The gut also has its own "blood-brain barrier" that helps maintain its physiological environment, often referred to as the "second brain." Recent research indicates that gut microbiota and their metabolites can impact brain activity.

As the most significant micro-ecological system in the human body, gut bacteria play a crucial role in the material and energy metabolism of the body, significantly impacting human health. These bacteria live attached to the gut of animals and aid in carrying out a wide range of physiological and biochemical processes 2. There are 1,000 to 1,500 different types of bacteria, or around 100 trillion, in the average human gut. It consists principally of Firmicutes and Bacteroidetes 3. Bacteroidetes mostly create propionic acid and butyric acid, whereas Firmicutes primarily make butyric acid 4. Faecalis dandelion is one of the most prevalent microbes in the colon, and its decrease may be utilized to identify gut disorders like as Crohn's disease and ulcerative colitis 5. Simultaneously, the gastrointestinal system is a critical organ in the production of metabolites and neuroactive chemicals. Recent studies have found that approximately 40% of human metabolites, including short-chain fatty acids, vitamins, and trimethylamine, are produced by the gut microbiome 6.

**Figure 1:**
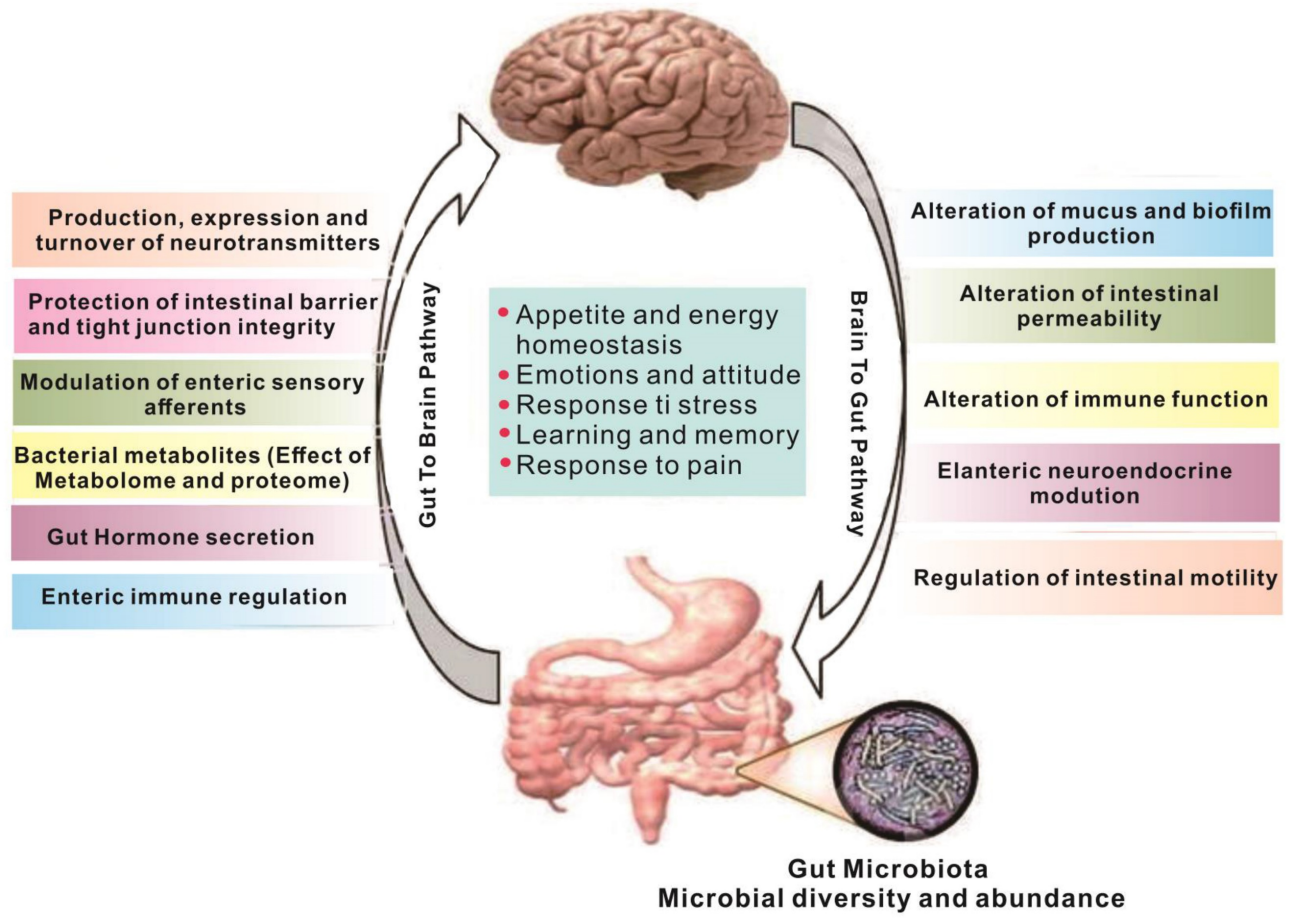
The gut and brain interact 11. Regulation between the gut and the brain is a two-way regulatory process, and the gut microbiome has a direct or indirect regulatory effect on the brain mainly through neuro-immune and neuroendocrine mechanisms. The nervous system can also regulate gut function, thereby modulating gut microbiota composition and activity.

Metabolites produced in the gut are closely linked to brain activity, and communication between the gut and brain occurs through the "gut-brain axis"^7^,^8^ - a two-way communication system involving neural, metabolic, endocrine, and immune pathway (Figure 1). This communication coordinates the body's functions through messengers such as neural, metabolic, endocrine, and immune mediators 9. Substances in the gut need to cross multiple barriers in the process of reaching the brain, the most important of which are gut mucosal barrier and blood-brain barrier. The gut mucosal barrier can separate the gut cavity from the internal environment of the body, avoid harmful substances such as bacteria and endotoxin in the gut cavity entering the body, and also block some drugs from entering the body to reduce the efficacy of drugs. Meanwhile, the blood-brain barrier regulates substance exchange between the central nervous system and the blood. A significant proportion of drugs are blocked by the blood-brain barrier and cannot enter the brain to treat diseases 10.

While gut-derived γ-aminobutyric acid (GABA) can enter the brain through the corresponding transporters on the blood-brain barrier 12, other neurotransmitters produced in the gut cannot enter the brain, even if they can pass through the gut mucosal barrier. However, they can indirectly affect brain function through the enteric nervous system, vagus nerve or by regulating the expression of peripheral receptors 13,14. The vagus nerve is particularly crucial in this regard, as it is a key component of the gut-brain axis. It is a complex network that connects the gut and the brain in both directions, receiving information from the gut and transmitting it to the brain.

As a result, this review aims to summarize the intricate relationship between gut microbial metabolites and the brain. The types of gut microbial metabolites, gut-brain signaling pathways, the effects of metabolites on brain cells, and brain diseases and treatment measures related to metabolites are all thoroughly explained. The review also highlights the crucial role of gut microbial metabolites in brain diseases and their treatment, providing viable treatment strategies for non-invasive brain diseases and paving the way for the continuous development of related fields.

## Relationship between gut microbe-driven metabolites and brain diseases

**Figure 2:**
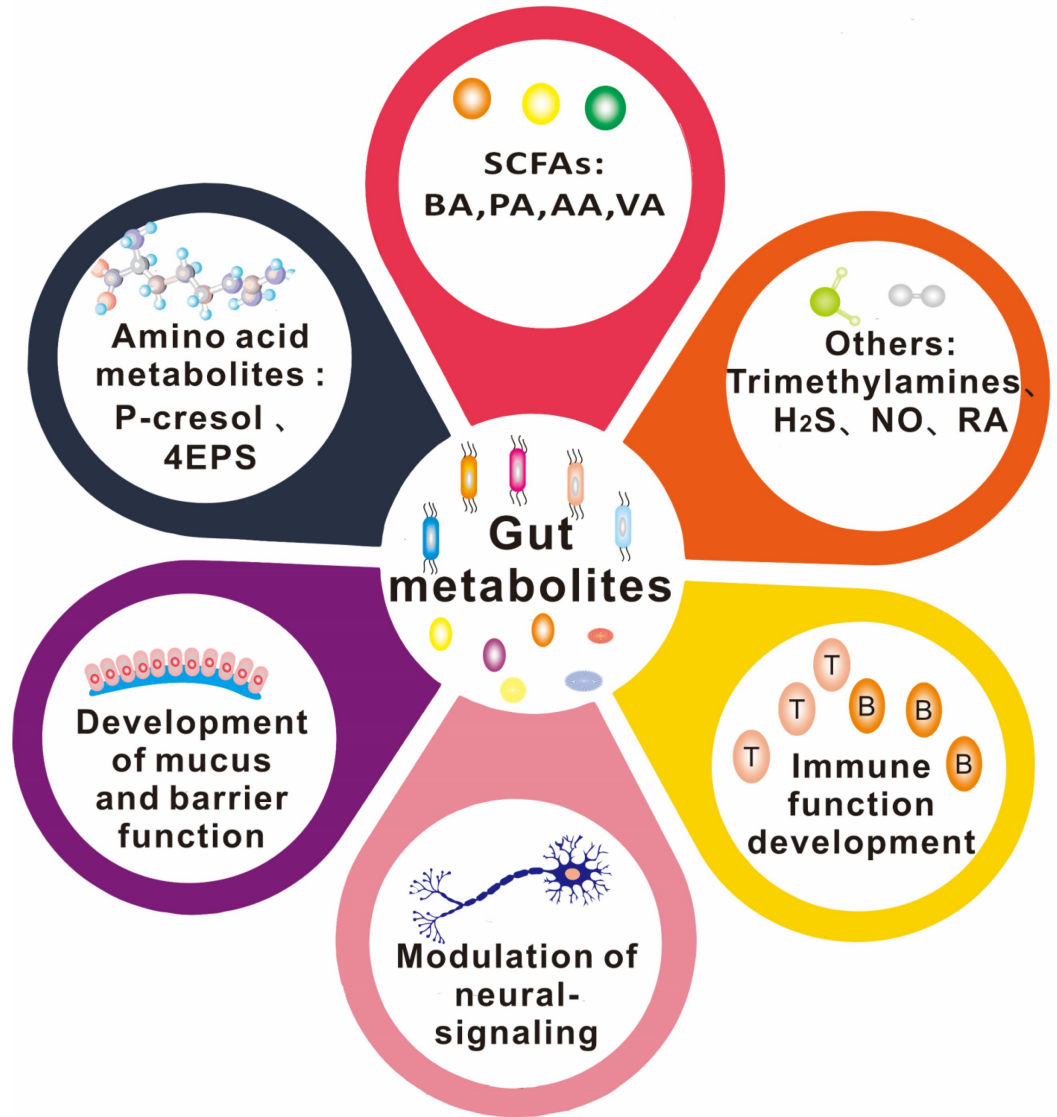
The types of gut metabolites and their physiological functions. Gut metabolites are mainly short-chain fatty acids, amino acid metabolites, complex plant polysaccharides, vitamins, and others such as TMAO (Trimethylamine-N-oxide) and some gas molecules. Its physiological role in the body is more diverse, such as SCFAs (short-chain fatty acids) were found to work on the integrity of the blood-brain barrier, through the brain-gut axis on the nervous system, regulating immune system function. 4-EPS (4-ethylbenzene sulfate), BA(butyric Acids), PA(propionic acid), AA(acetic acid), VA(valeric acid).

**Figure 3:**
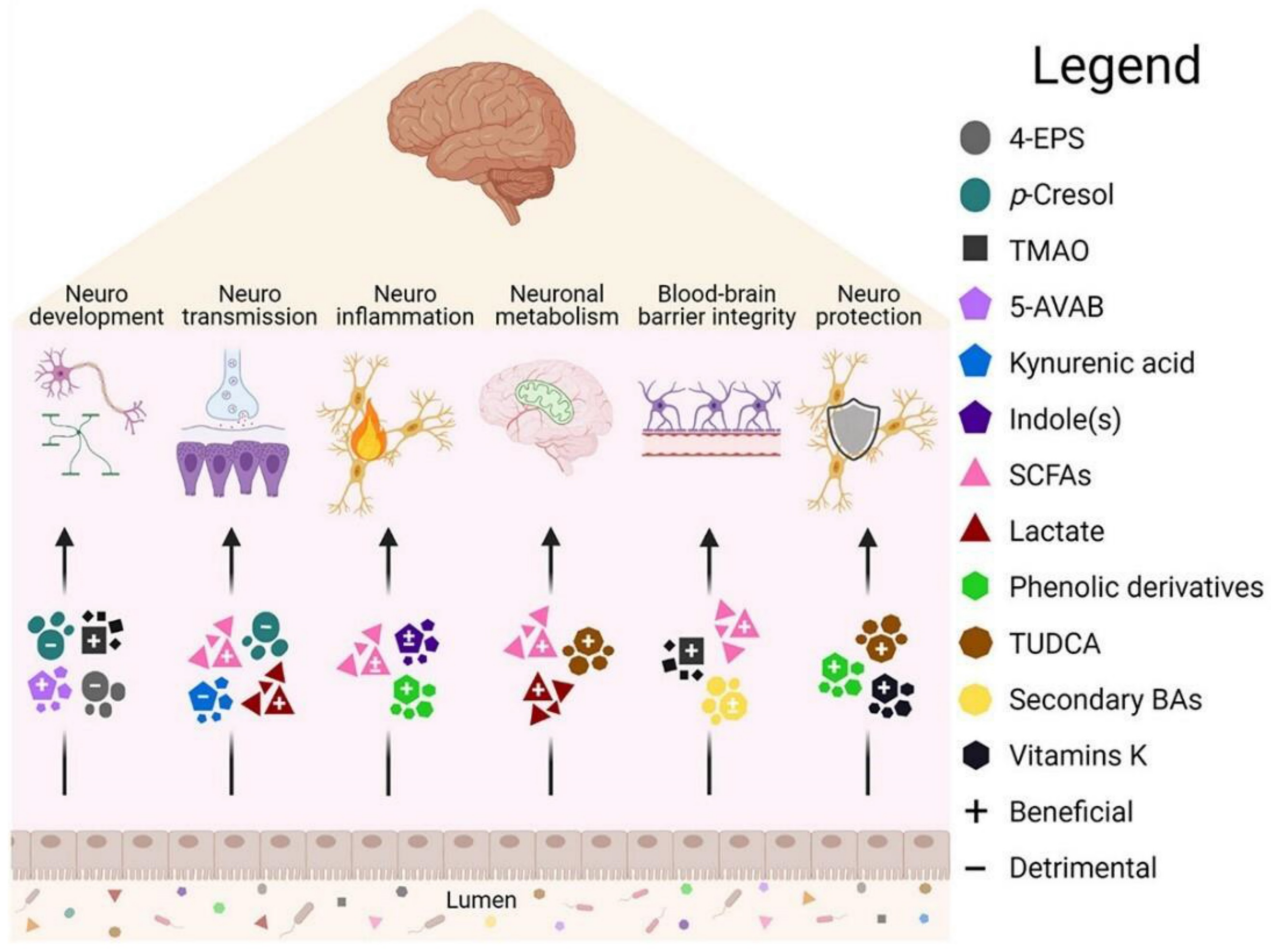
Brain function is affected by intestinal metabolites 15. Some metabolites in the intestine have been shown to have beneficial (+) or harmful (-) effects on the body, but their effects depend on the pathway of action and disease models., 5-AVAB (5-aminovaleric acid betaine), BA (Bile acid), TUDCA (Taurine deoxycholic acid).

There is a growing body of evidence supporting the important role of gut microbe-driven metabolites in the development and progression of brain diseases. Short-chain fatty acids (SCFAs) such as acetate, propionate and butyrate, produced by gut microbes, have been shown to regulate the blood-brain barrier, affect neuroplasticity, and modulate the maturation of microglia. The metabolites produced by the microbiota, including short-chain fatty acids, trimethylamine, and amino acid metabolites, among others, take many different forms, which are further classified and elaborated upon below. Figure 2 and Figure 3 illustrate the types and functions of microbiota-mediated metabolites in the gut. Table 1 lists the types of gut metabolites and their related microorganisms and the diseases they cause, especially brain diseases. Changes in the composition of gut microbe-driven metabolites can lead to impaired innate immunity in the brain, which is associated with various brain diseases. Thus, understanding the role of gut microbe-driven metabolites in brain function and disease has become an important research focus, with potential implications for the development of novel therapeutics for brain diseases.

### Short Chain Fatty Acids (SCFAs)

Fatty acids are carboxylic acids with aliphatic tails of varying lengths, and SCFAs are generally defined as carboxylic acids with aliphatic tails of fewer than 6 carbons 16. Substantial evidence now suggests that SCFAs play a crucial role in maintaining health and disease progression. In humans, SCFAs are mainly produced by microbial fermentation of dietary fiber in the cecum and colon, yielding a variety of SCFAs, including acetate, propionate, and butyrate. Although acetate is the most abundant SCFAs in the body, butyrate is present at high levels in the gut lumen and serves as the primary energy source for colon cells. Moreover, butyrate can regulate neuroplasticity, epigenetics, and gene expression, affecting the maturation of microglia and acting as a signaling molecule in the central nervous system.

Furthermore, SCFAs can affect the physiological structure of the brain by regulating gut permeability through modulating tight junction proteins that are also components of the blood-brain barrier. Thus, there is evidence that SCFAs may also affect the integrity of the blood-brain barrier. Studies on sterile mice have shown that SCFAs can indeed regulate the permeability of the blood-brain barrier, thereby influencing the extent to which beneficial or harmful molecules from the circulation reach brain tissue. For example, during the body's development, SCFAs can strengthen the blood-brain barrier and reduce its permeability to harmful factors such as inflammatory molecules, thus protecting the brain from damage. Alterations in the gut microbiota composition of sterile mice affected the maturation of microglia in the brain, resulting in impaired innate immunity. However, oral administration of SCFAs restored morphological changes in microglia.

Additionally, SCFAs can also impact the body's behavior and mood. For instance, sodium butyrate as a histone deacetylase inhibitor, can induce temporary acetylation of histones in the frontal cortex and hippocampus of mice after injection into depression model mice. Furthermore, there was also a dynamic change in the expression of brain-derived neurotrophic factor (BDNF), a gene involved in the behavioral and pharmacological model of depression. After long-term administration of sodium butyrate, the depressive behavior of mice significantly reduced 17. Oral administration can also reduce the sensitivity of PTZ-induced epilepsy, but only in mice with colitis and increased seizure sensitivity 18. Amyotrophic lateral sclerosis (ALS) can affect neurons in the brain and spinal cord, leading to premature death of motor neurons. Patients typically die from respiratory paralysis within 3-5 years. Numerous studies have found that patients with ALS have abnormal gut microbiota, and the number of bacteria producing butyrate is reduced 19. Moreover, the concentration of short-chain fatty acids in fecal samples of patients with Parkinson's disease is significantly reduced 20, particularly propionate. In another study, the concentration of SCFAs in serum of Parkinson's patients and normal people was measured. The levels of propionic acid, butyric acid and caproic acid in Parkinson's patients were lower than those in normal people, while the level of heptanoic acid was higher than that in the control group.

In addition, it has been found that changes in propionic acid level are related to the symptoms of Parkinson's patients. After taking the treatment drugs, the serum concentration of propionic acid recovered, indicating that SCFAs may affect the symptoms of Parkinson's patients through the bloodstream 21. When the gut microbiota is disrupted, the level of SCFAs, such as butyrate, that promote the upregulation of brain-derived neurotrophic factors decrease, which can affect neuronal development and pathological conditions 7. The severity of disease in patients with autism spectrum disorders (ASD) is influenced by an increase in propionate-producing bacteria and a decrease in butyrate-producing bacteria 22. This has been confirmed by researchers who applied propionate to rodents in various ways to construct autism models 23, and sodium butyrate can enhance the social ability of autistic mice 24. When propionate was applied to mice through different pathways, the activation of microglia was significantly increased, inducing autism-like behavior in the mice. However, butyrate has an anti-inflammatory effect in microglia, which promotes transcription of genes involved in the neuronal inhibitory pathway, thereby improving the behavioral state of mice with an autistic spectrum idiopathic model 25.

Furthermore, the role of SCFAs in cardiovascular diseases cannot be ignored. Bartolomaeus and his team found that propionate can significantly reduce myocardial hypertrophy, fibrosis, vascular dysfunction, and hypertension by studying the role of propionate in a hypertensive mouse model 26. Chen and his colleagues also found that propionate can slow down the pathogenesis of cardiovascular disease, When the gut microbiota composition of rats fed with high-salt diet changes, which in turn affects the production of propionate, the occurrence of hypertension is greatly promoted 27. Stroke can alter the composition of gut microorganisms and vice versa. By regulating the composition of gut microbes in stroke patients, which in turn alters the production of gut metabolites, stroke-related symptoms can be improved. Rebecca and his colleagues found that supplementing SCFAs to stroke model mice significantly improved the motor function of the affected limb. Further analysis showed that it was related to the improvement of microglia activation by SCFAs 28. Cognitive impairment after stroke is one of the common complications and seriously affects the patient's self-care ability in daily life. SCFAs have a certain protective ability 29. In addition, SCFAs play an important role in the regulation of peripheral inflammation. Vinolo and his team gave rats the butyric acid precursor drug tributyrin and found that it can reduce the production of pro-inflammatory cytokines such as NO and TNF-α by neutrophils in rats. It can also inhibit the activation of nuclear factor κB (NF-κB) and histone deacetylase (HDAC). The mechanism may be related to the weakening of LPS response 30.

Finally, SCFAs also affect the tumor growth in the digestive tract system by inhibiting HDACs preventing the occurrence of colorectal cancer and related inflammation 31. When the gut ecology of the body is out of balance and the bacteria that produce SCFAs are reduced, the level of SCFAs in feces decreases, making it easy to induce inflammatory bowel disease. At the same time, SCFAs have a direct impact on colon cell proliferation and differentiation to prevent colon cancer 32.

SCFAs play a crucial role in the brain, and their levels have been found to be altered in various neurological disorders, including Parkinson's disease and Alzheimer's disease. Moreover, it has been suggested that SCFAs may also contribute to the pathogenesis of these diseases. These findings highlight the potential importance of SCFAs in the development and progression of brain diseases, and offer a new perspective for future therapeutic interventions.

### Trimethylamines

**Figure 4:**
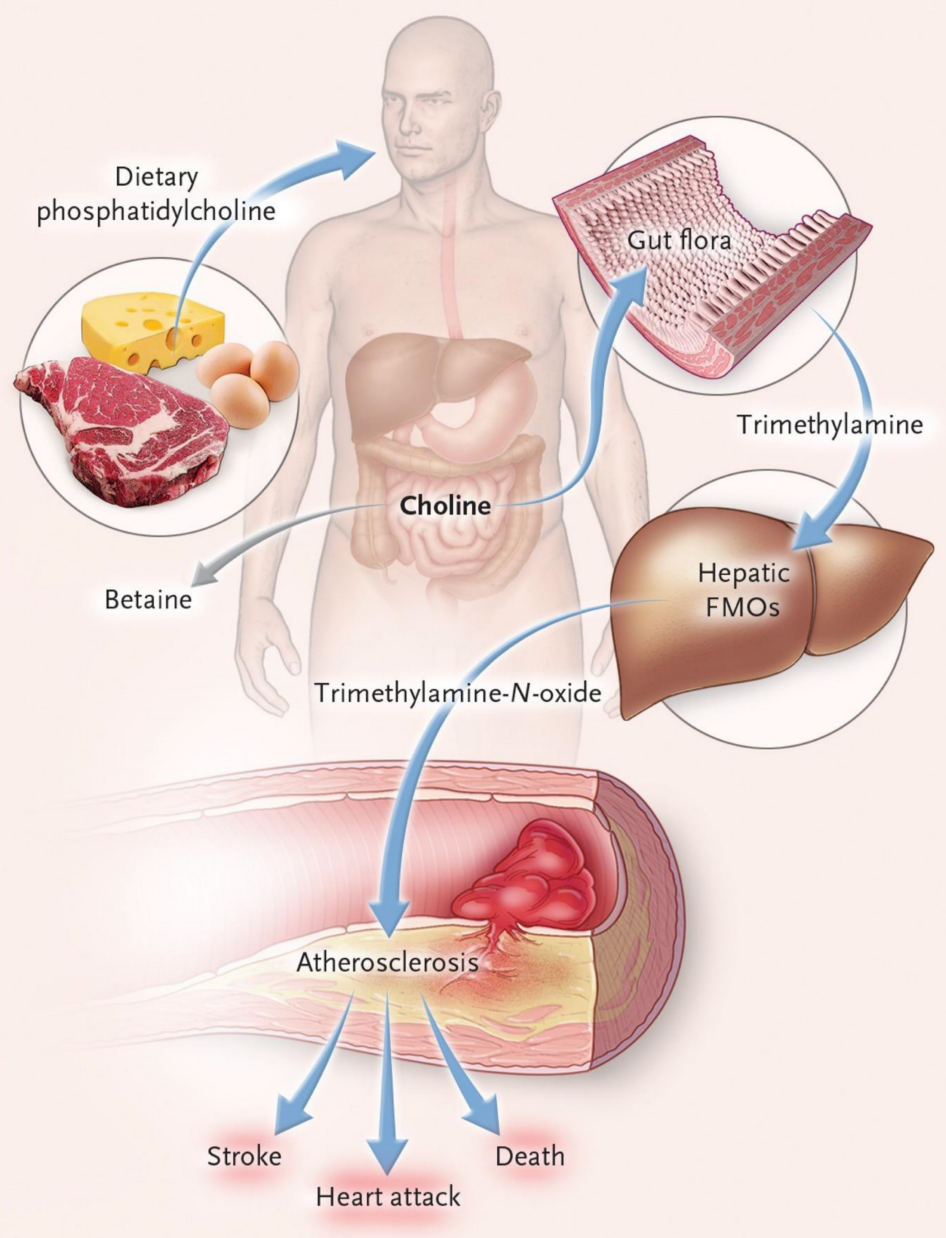
The connection of dietary phosphatidylcholine, gut microbiota, and incident adverse cardiovascular events^41^. Ingested phosphatidylcholine is first metabolized by gut microbes to trimethylamine (TMA). TMA is rapidly further oxidized to trimethylamine-N-oxide (TMAO) by hepatic flavin-containing monooxygenases (FMOs) and increased risk of heart attack, stroke, and death.

Trimethylamine is a precursor of trimethylamine-N-oxide (TMAO), which is produced by the metabolism of nutritional precursors such as choline, phosphatidylcholine and L-carnitine by gut microorganisms. The conversion of trimethylamine to TMAO occurs in the host liver FMO3 (flavin-containing monooxygenase 3) (Figure 4). TMAO is a gut microbiota metabolite that promotes atherosclerosis and thrombosis 33. Elevated level of TMAO in the blood may be an important prognostic factor for adverse cardiac events in patients with chronic heart failure after myocardial infarction 34. In patients with heart failure, gut microbiota translocation can occur, allowing gut microbial metabolites to enter the blood circulation through the damaged gut barrier, which may cause local or systemic inflammatory responses. Among these metabolites, TMAO can aggravate the progression of heart failure by inducing myocardial hypertrophy and fibrosis, and its increased level in the blood can also lead to incident atrial fibrillation 35. In patients with hypertension, Nie and his team showed, through clinical data analysis, that TMAO levels were positively correlated with the incidence of stroke during treatment 36. Yin and his colleagues also conducted a case-control study in asymptomatic patients with clinical atherosclerotic ischemic stroke and transient cerebral ischemia, and found that patients with gut opportunistic pathogens such as enterobacter increased, while beneficial symbiotic bacteria decreased, leading to decreased blood TMAO levels 37.

A long-term study conducted by Yoriko and his team measured TMAO levels in the blood of 760 healthy women who had drawn blood twice at 10-year intervals. Among them, patients with coronary heart disease patients (n=380) had no difference in TMAO levels compared to the control group (n=380) at the first blood draw, but higher plasma TMAO levels were found at the second blood draw 38. Lumbar punctures were performed on 58 patients with different neurological disorders to obtain cerebrospinal fluid, which demonstrated that TMAO could be evaluated in cerebrospinal fluid, but its pathophysiological role in the central nervous system needs further study 39. In mouse studies, it was found that TMAO increased the number of senescent cells, mainly neuronal cells, which could induce brain aging and age-related cognitive impairment in mice and promote brain aging in mice 40.

The concentration of TMAO in the cerebrospinal fluid of patients with Alzheimer's disease has been found to significantly increase 42. Additionally, it can serve as a biomarker for early Parkinson's disease. Seok and his team measured plasma TMAO in 85 early PD patients and 20 healthy people. The plasma TMAO content of PD patients was lower than that of the control group, and individuals with high levels of TMAO had a lower risk of dementia transformation 43. TMAO also plays a significant role in the progression of osteoporosis, with levels in the body being negatively correlated with the bone mineral density. This is due to its ability to activate the NF-κB signaling pathway, thereby regulating the function of bone marrow mesenchymal stem cells and accelerating bone loss and the progression of osteoporosis 44. However, TMAO also has beneficial effects on the body, including reducing endoplasmic reticulum stress and lipogenesis in adipocytes, increasing insulin secretion, and mitigating the body's glucose tolerance caused by diet 45.

### Amino acid metabolites

The presence of tryptophan is essential for protein synthesis in the human body and it plays a crucial role in the microbe-gut-brain axis. As the solo precursor of the neurotransmitter serotonin, tryptophan is involved in various human activities. Its metabolic pathways primarily include the kynurenine pathway and 5-HT pathway, and it can also be directly converted into various of compounds. Depending on its metabolic pathway, tryptophan can produce different metabolites that impact the occurrence of depression 46. Metabolites of tryptophan produced via the kynurenine pathway regulate neural activity and play a vital role in the onset and improvement of brain and nervous system disorders 47. In the gut, microorganisms metabolize tryptophan into five molecules: indole-3-aldehyde, indole-3-acetic acid, indole-3-propionic acid, indole-3-acrylic acid and indole-3-acetaldehyde. All of these molecules are aromatic receptor ligands that can prevent the activation of astrocytes and microglia by blocking pro-inflammatory transcription factors, thereby mediating brain homeostasis 48, among them, indole-3-acetic acid also plays a role in the negative effect of high-tryptophan diet on energy balance, thereby reducing the weight gain of rats.

Gut bacteria containing glutamate racemase, such as corynebacterium Glutamicum, have the ability to convert L-glutamic acid into D-glutamic acid, which can affect N-methyl-D-aspartate (NMDA) glutamate receptors and cognitive function in patients with Alzheimer's disease. NMDA glutamate receptor enhancers have been found to improve the cognitive function of patients with Alzheimer's disease and dementia 49. Phenols are metabolites of tyrosine produced by gut microorganisms 50. Elevated levels of gut microbial metabolite P-cresol have been detected in the urine and feces of autistic patients, and it has been found that the severity of behavioral changes in autistic patients is correlated with urinary P-cresol levels 51. Overproduction of the aromatic amino acid metabolite indole can also cause anxiety and depression 52. Urea and asymmetric dimethylarginine are circulating nitrogen metabolites that are also features of chronic kidney disease and coronary heart disease 53,54. Serum indoxyl sulfate levels are considered markers of coronary atherosclerosis in clinical practice 55. In addition, indole can counteract the harmful effects of lipopolysaccharide in the liver, thereby reducing liver inflammation 56. Indole can also regulate the body's sense of hunger, stimulate vagal afferent activity, and induce colon endocrine L cells to produce the corresponding response to the secretion of GLP1 (Glucagon-like peptide 1) regulate the sense of hunger 57.

### Vitamins

Robert summarized and discussed the important role of vitamins in the gut microbiota. Although the human body obtains most of its vitamins from daily diets, the gut microbiota is also an important source of vitamins, such as vitamin C, vitamin K, and B vitamins 58. B vitamins are necessary cofactors for many metabolic pathways in the human body, including the metabolism of neurotransmitters. They are involved in myelination and neuroprotection processes and play an anti-inflammatory and antioxidant role 59. Therefore, the lack of B vitamins often leads to neurological abnormalities, which are manifest as mental disorders. For instance, vitamin B_3_ deficiency is often associated with brown skin disease, accompanied by dementia, delirium and other symptoms 60.

Retinoic acid (RA) is a product of vitamin A metabolism by gut epithelial cells, which regulates the development of protective and pathogenic immune responses in the gut in a concentration-dependent manner. However, gut microbiota such as Clostridium can reduce the level of retinol in the gut by inhibiting the expression of retinol dehydrogenase 7. By comparing the levels of vitamin A-related metabolites in the gut and liver of sterile mice and normal mice, it was found that the content of vitamin A active metabolites in the gut and the storage of vitamin A in the liver 61. Moreover, the gut microbiota regulates the body's immune function by regulating vitamin A metabolism 62.

### Complex plant polysaccharides

Dietary polysaccharides are the main source of energy for mammalian gut microbiota. However, most polysaccharides cannot be directly digested and absorbed after entering the digestive tract. Herbivores get 70% of their energy from these polysaccharides due to the role of gut microbes in breaking down the polysaccharide polymers 63,64. The gut microbiota encodes a large number of carbohydrate-active enzymes, which make the polysaccharide into secondary metabolites or fermentation products for human absorption 65.

Plant polysaccharides are believed to have various pharmacological activities, such as regulating immune function, anti-tumor, anti-aging effects. They also affect the gut environment after entering the gut. For example, they increase the production of acidic substances in the gut through fermentation, which reduces the gut pH value and affect the composition of the microbiota and the balance of microbial metabolites. This can indirectly increase the number of bacteria producing butyrate and the level of butyrate 66, which can then indirectly affect the physiological and pathological activities of the body. Additionally, plant polysaccharides can act as prebiotics through their metabolites, thereby regulating the gut microbiota and restoring the diversity of gut ecology 67.

### Others

Apart from the gut microbiota metabolites discussed above, other metabolites in the body play an important role in physiological and pathological activities. Polyphenols, urolithins, and some gas molecules are some examples. Polyphenols can promote memory, learning and cognitive abilities by protecting neurons and inhibiting neuroinflammation. They protect the brain both directly and indirectly. The direct effect is their ability to cross the blood-brain barrier 68. And studies have shown that polyphenol metabolites can enter the brain through the blood-brain barrier to play a protective role 69. The indirect effect is through improving peripheral cerebrovascular health 68. Research indicates that polyphenols can improve vasodilatation and increase circulating NO levels 70. Additionally, gut microbes can produce some physiologically active gas molecules such as H_2_S, NO and CO, which are generally associated with cardiovascular disease 71.

Tannins can be metabolized by gut microorganisms to produce urolithin A, B, and C. Urolithin A has been shown to inhibit osteoporosis by enhancing the autophagy ability of bone marrow macrophages and has the opposite effect of TMAO 72. Urolithin B can prevent myocardial cell apoptosis caused by ischemia and hypoxia, promote nerve remodeling after hypoxia, reduce the susceptibility of myocardial arrhythmia 73, and inhibit the accumulation of advanced glycation end products in aging model mice to improve learning and memory function 74. On the other hand, L-carnitine can be metabolized by gut microbes to produce crotonic acid, which can promote atherosclerosis 75. In addition, excessive production of oxidants in the body can lead to cell dysfunction and many diseases, but antioxidants produced by the body can eliminate the damage caused by excessive oxidants. The active sulfur species is a newly discovered strong antioxidant that can significantly enhance the body's antioxidant activity through the gut microbial metabolism of sulfur in the gut.^76^.

## Routes of gut microbe-driven metabolite to brain

**Figure 5:**
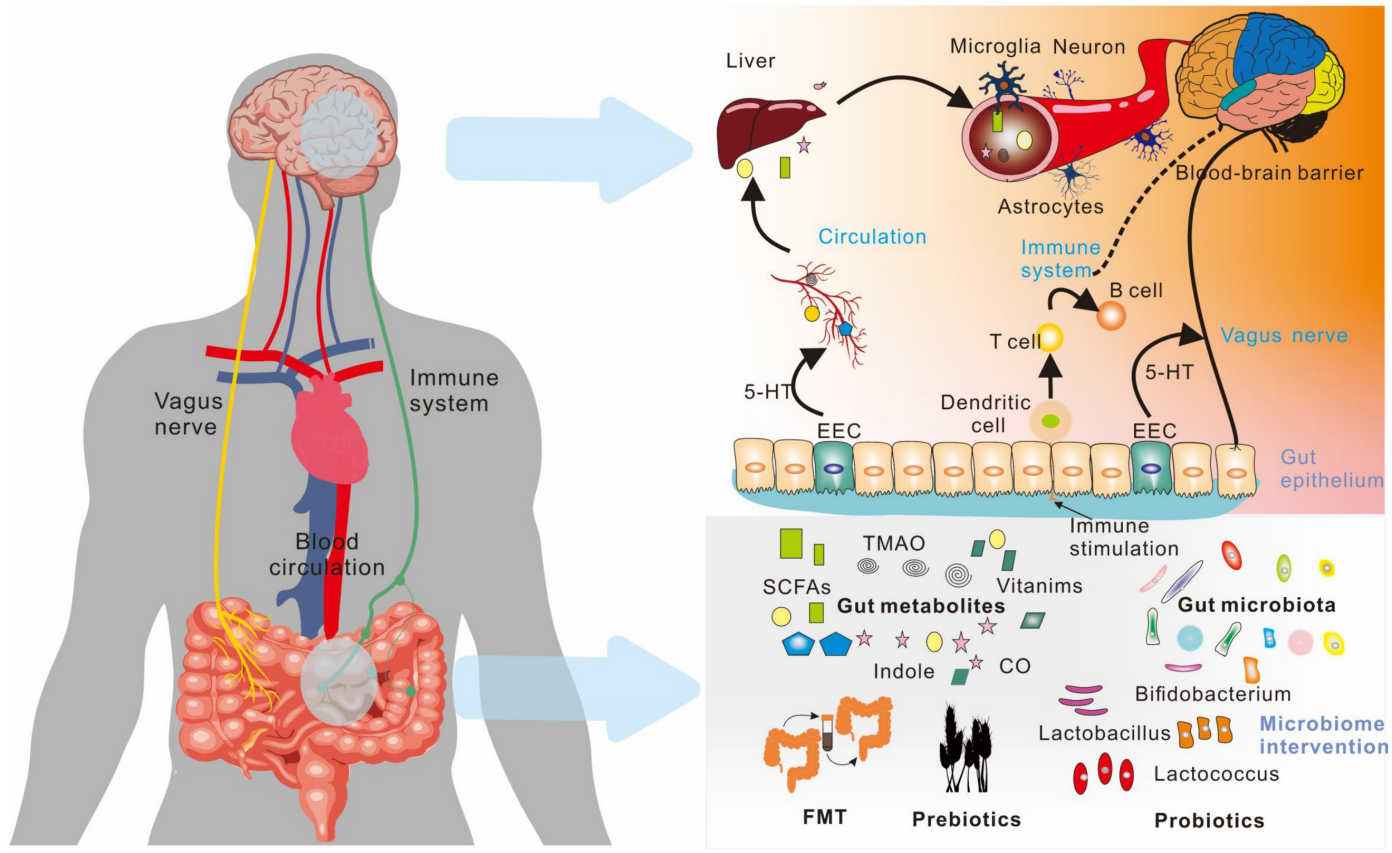
Microorganism-gut-brain axis. The communication between the gut and the brain is two-way through the gut-brain axis, including the vagus nerve, the immune system, and blood circulation. Various metabolites produced by the gut microbiota play an important role in these pathways.; EEC (Enteroendocrine Cell); FMT (Fecal microbiota transplant).

Various neurological, neurodegenerative, and neuropsychiatric illnesses have been linked to changes in the composition of the gut microbiota. Microbes in the gut transform and metabolize chemicals from food and the host to produce various metabolites with both local and systemic effects. These metabolites have the potential to affect the normal function of the body, particularly the brain. This chapter provides a comprehensive analysis of the metabolic pathways that lead to the brain via "gut-brain axis". The "gut-brain axis" is a complex system that involves the blood, immune system, and vagus nerve, and facilitates communication between the brain and the metabolites produced by the gut microbiota. The following sections provide detailed descriptions of these routes of gut microbe-driven metabolites to the brain (as shown in Figure 5).

### Blood circulation

The role of blood circulation is to maintain the normal metabolism of the body and transport metabolites, both beneficial and harmful, to various parts of the body, including the brain. Studies have shown increased peripheral regulatory T cell production in mice after oral administration of butyrate and propionate 120. SCFAs can also regulate monocytes in the mouse brain, which can affect neural structure and function, as well as advanced brain function once they reach the bloodstream and the brain 121. Furthermore, functional SCFAs receptors are present in the central and peripheral nervous systems, and that FFAR3 receptors are highly expressed in rat brain tissue 8, suggesting that SCFAs may affect the central nervous system by regulating the secretion of neurotransmitters such as GABA and 5-HT 67.

### Vagus nerve

The vagus nerve plays a crucial role in the communication between the gut and the brain. It is composed of 80 % of the afferent nerves and 20 % of the efferent nerves, which directly connect the brain and gut and govern almost all digestive tracts. However, the afferent fibers of the vagus nerve do not directly contact the gut microbiota and contents, but transmit signals through bacterial metabolites 122. As the main pathway connecting the gut and the brain, the vagus nerve is the fastest and most direct way of communication between the two. Metabolites produced by microorganisms in the gut lumen transmit information directly or indirectly to the brain through the humoral and neuronal pathways of the vagus nerve 123. The regulation of neurotransmitter levels in the brain by electrical stimulation of the vagus nerve can be used to treat epilepsy and depression 124, Gut bacteria-related products such as LPS can also activate vagus nerve afferents at the myelin level of the vagus nerve, thereby affecting brain function 125. Feeding Lactobacillus rhamnose can regulate the neuroemotional behavior of mice and the expression of GABA receptors in the brain. However, this change disappears in mice undergoing vagotomy, indicating that the regulation of the brain of mice after ingestion of Lactobacillus is carried out through the vagus nerve 13.

**Figure 6:**
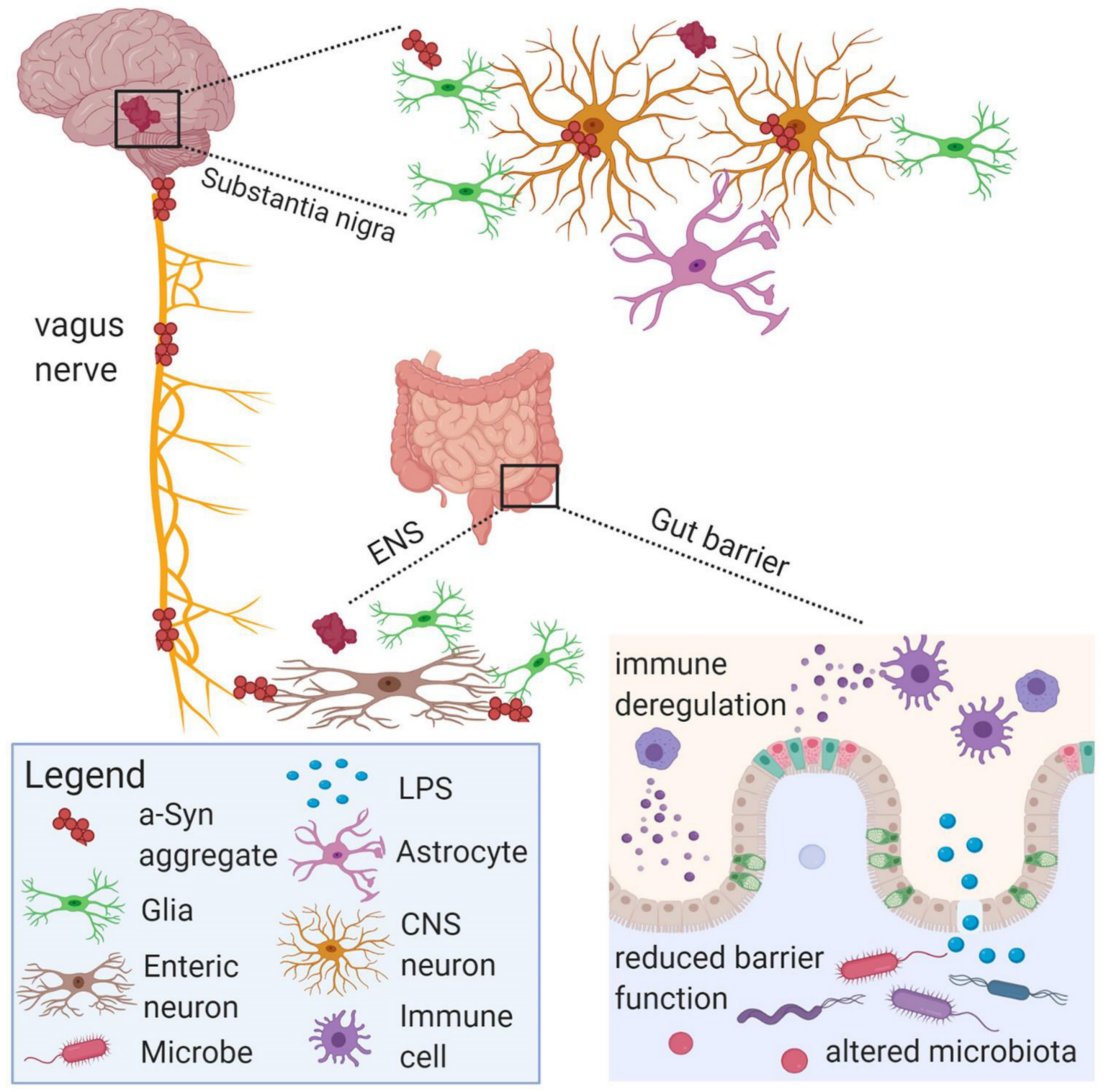
The gut microbiota and its metabolites can communicate with the vagus nerve through a special structure called neuropods on enteroendocrine cells, and some harmful substances such as LPS and α-Syn can spread pathologically through the vagus nerve to damage central nervous system cells and lead to central nervous system diseases 129.

Similarly, the beneficial effect of Bifidobacterium longum treatment on improving anxiety-like symptoms caused by chronic gut inflammation disappear after destroying the integrity of the vagus nerve 126. Intraperitoneal injection of SCFAs in mice inhibited food intake, but this effect was attenuated after vagotomy 127. SCFAs can also directly activate the afferent activity of the vagus nerve. For instance, sodium butyrate is applied to anesthetized rats to induce afferent discharge of the vagus nerve. The mechanism of action is not related to the CCK-A receptor present on the vagus nerve, but directly acts on the vagus nerve 128. Figure 6 illustrates how gut microbial metabolites affect central nervous system cells via the vagus nerve.

### Immune system

Gut microbiota plays a crucial role in maintaining host homeostasis by producing a diverse array of metabolites that have far-reaching effects on various aspects of host physiology, including food metabolism and immune system function.^130^. Recent studies have demonstrated that these metabolites have a profound impact on brain development and function, with the immune system playing a key role in mediating these interactions. Microbial metabolites can affect not only immune cells in the gut, but also those in the brain. Changes in neurogenesis, plasticity, and responses to neuroinflammation may involve immune system activation in both the gut and the brain. Disruptions in this bidirectional communication, as seen in dysbiosis, have been linked to the development of psychiatric and neurodevelopmental disorders such as autism spectrum disorders in patients with gastrointestinal diseases such as inflammatory bowel disease. Therefore, probiotics, including beneficial bacteria and microbial metabolites, have gained attention as potential therapeutic targets for influencing behavior and cognitive development 131.

### Other (Enterochromaffin cell stimulation)

Enterochromaffin cells are a type of gut endocrine cells, which are small in size and number. They express an electrically excitatory chemoreceptor, that connects 5-HT-sensitive afferent nerve fibers through synapses, thereby transmitting gut signals to the nervous system. SCFAs can promote the transcription of tryptophan hydroxylase in human enterochromaffin cell models and increase the synthesis of 5-HT in cells 132. Studies have also shown that enterochromaffin cells not only have an indirect effect on the brain through serotonin but can also be indirectly linked to neurons. Substance P (SP) is a sensory neurotransmitter that was originally found in the central nervous system but was also found in enterochromaffin cells in subsequent studies. Further studies concluded that SP is produced, stored and secreted by gut chromaffin cells 133. Stimulation of enterochromaffin cells can have a regulatory effect on the vagus nerve, which subsequently affects the brain. As a result, enterochromaffin cells function not only as a bridge but also as an essential component of the gut-brain axis 134.

## Gut microbe-driven metabolite affects brain diseases through brain cells

**Figure 7:**
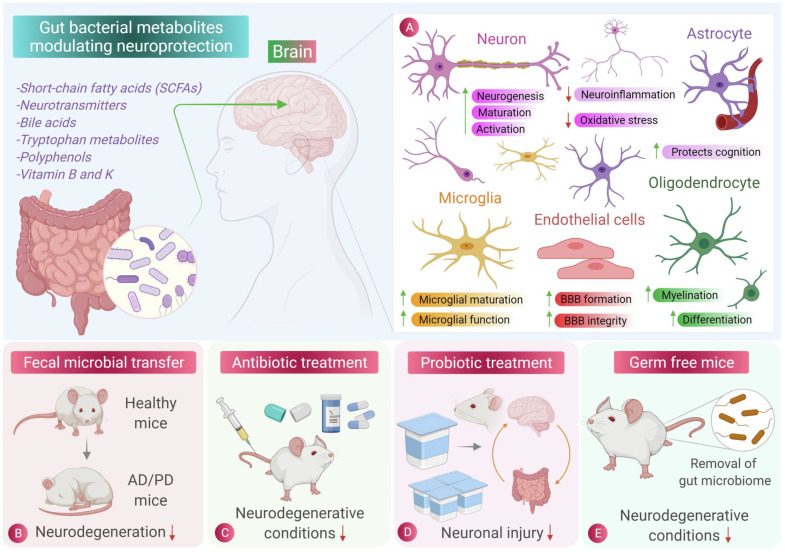
Role of gut microbiota and its metabolites in central nervous system cell protection 135. The beneficial metabolites produced by intestinal microbes can reduce inflammation and oxidative stress in central nervous cells. Fecal microbiota transplantation, antibiotic therapy and probiotic therapy have inhibitory effects on the occurrence and progression of neurodegenerative diseases such as Parkinson's disease and Alzheimer's disease. Sterile mice showed a lower incidence of neurodegenerative diseases, indicating the role of intestinal flora and its metabolites in neurodegenerative diseases.

Recent studies have revealed the crucial role of gut microbe-driven metabolites in the development and progression of brain diseases. These metabolites can influence the normal functioning of brain cells by altering their morphology and synaptic plasticity, resulting in cognitive dysfunction and other neurological symptoms (as shown in Figure 7). Overall, these findings underscore the significance of gut microbe-driven metabolites in maintaining brain health and indicate that targeting these metabolites could represent a novel therapeutic strategy for brain diseases. In the following sections, we will provide a detailed overview of gut microbe-driven metabolites that impact brain diseases via brain cells.

### Neurons

Neurons are the fundamental units of the central nervous system, responsible for receiving and transmitting electrical and chemical signals. Neurological disorders, such as epilepsy and schizophrenia, can result from neuronal hyperexcitability or other abnormalities.

Recent studies have shown that gut microbial metabolites can affect neuronal function and play a role in the development and treatment of neurological diseases. For example, S-equol, a gut microbial metabolite of soybean isoflavones, has been found to improve TMEV-induced neuronal hyperexcitability in mice by modulating the gut microbiota.^136^. Intermittent fasting has also been shown to promote axonal regeneration through the increase of Gram-positive bacteria and the gut microbial metabolite indole-3-propionic acid (IPA) in serum.^137^. Urolithin A, a metabolite of ellagic acid-containing food in the gut, has a protective effect in Parkinson's disease by promoting mitochondrial biogenesis through the SIRT1/PGC-1α neural pathway 138.

These findings suggest that gut microbial metabolites may have potential as novel therapeutic targets for the treatment of neurological diseases.

### Astrocytes

Astrocytes play a vital role in maintaining the homeostasis of the central nervous system. Dysfunctional astrocytes have been linked to various neurological and mental disorders 139. The gut microbiota can indirectly regulate the function of astrocytes through immune activation and inflammatory mediators and cytokines. For instance, gut microbial metabolites like SCFAs, 5-HT and aryl hydrocarbon receptor ligands can stimulate gut epithelial cells and macrophages in the gastrointestinal tract, leading to the activation of immune response and the production of inflammatory cytokines. These cytokines can reach the brain through the blood-brain barrier via systemic circulation and regulate the functions of astrocytes 140.

Studies have shown that long-term Lactobacillus enteral treatment in rats can cause changes in the central nervous system. In rats treated with enteral treatment for six months, the reactivity of astrocytes continuously decrease, affecting their development 141. Lactobacillus in the gut has the ability to produce GABA, which can enter the brain to activate the GABA receptor of astrocytes, causing the body to exhibit attentional disorders and anxiety-like behaviors 142. *In vitro* experiments have also showed that sodium butyrate and indole-propionic acid can prevent LPS-induced elevation of cytokines and kynurenine levels in human primary astrocytes 143.

However, the activation of astrocytes and microglia is the primary cause of neuroinflammation and neuronal damage. In Parkinson's disease mice, fecal butyrate content is much higher than in normal mice, leading to increased activation of microglia and astrocytes and neuroinflammation. Researchers have also fed a mixture of SCFAs to Parkinson's mice and found similar results. Thus, while some gut microbial metabolites can have beneficial effects on astrocytes, others can lead to neuroinflammation and neuronal damage 144.

### Oligodendrocytes

The specialized membrane of oligodendrocytes, called myelin, is critical for proper neural function. One mechanism by which the microbiota-gut-behavior axis affects behavior is by regulating myelin formation in the prefrontal cortex. In a study, butyrate was used to treat mice with demyelination induced by PA. The findings showed that butyrate improved demyelination and promoted the differentiation of immature oligodendrocytes, suggesting a potential therapeutic approach for myelin-related neurological disorders 145. Furthermore, studies have reported increased levels of the microbial metabolite 4-ethylphenyl sulfate (4EPS) in mouse models of atypical neurodevelopment. The gut microbiota can mediate the conversion of dietary tyrosine to 4-ethylphenol (4EP), which is then selectively metabolized to 4EPS. Its entry into the brain can cause oligodendrocyte damage in mice and cause anxiety-like behavior in mice, highlighting the importance of understanding the role of gut microbiota in neurodevelopmental disorders 146.

### Endothelial cells

Endothelial cells (EC) are monolayer cells that line the interior of blood vessels, and their nutritional and biochemical functions play a crucial role in cardiovascular function. Endothelial cell dysfunction is now recognized as a hallmark of most cardiovascular diseases, including microangiopathy associated with neurodegenerative diseases in diabetic patients. TMAO activates the mitogen-activated protein kinase (MAPK) pathway and induces EC dysfunction in vascular smooth muscle cells through NF-κB signaling, leading to upregulation of inflammatory signals and increased leukocyte adhesion to ECs. This aggravates endothelial dysfunction, reducing the ability of ECs to self-repair and increasing monocyte self-adhesion. These effects have been demonstrated *in vitro* experiments 147.

### Microglia

Microglia is resident immune cell in the brain, which can control innate immune function, and is crucial to the growth and development of the brain 22. Microglial dysregulation often in a series of psychiatric disorders 148. It has been confirmed that the gut microbiota can affect the maturation and function of microglia through its metabolites. Clostridium microorganisms can metabolize p-cresol, which can induce microglia activation and expression of microglia-associated CD68 protein. The microbial metabolites of Clostridium butyricum are mainly butyrate, which can reduce the activation of microglia and microglia-mediated neuroinflammation. Bacteroides can metabolize and produce propionate, inducing microglia activation and inflammatory mediators at high concentrations 25. Erny and his team demonstrated that immature microglia in germ-free mice were able to gradually mature through supplementing with SAFAs 149. Equol is a gut metabolite of dietary daidzein, and found that equol could attenuate microglia activation and enhance neuroprotective effects *in vitro* 150.

## Application of gut microbes-mediated metabolites in the treatment of brain diseases

**Figure 8:**
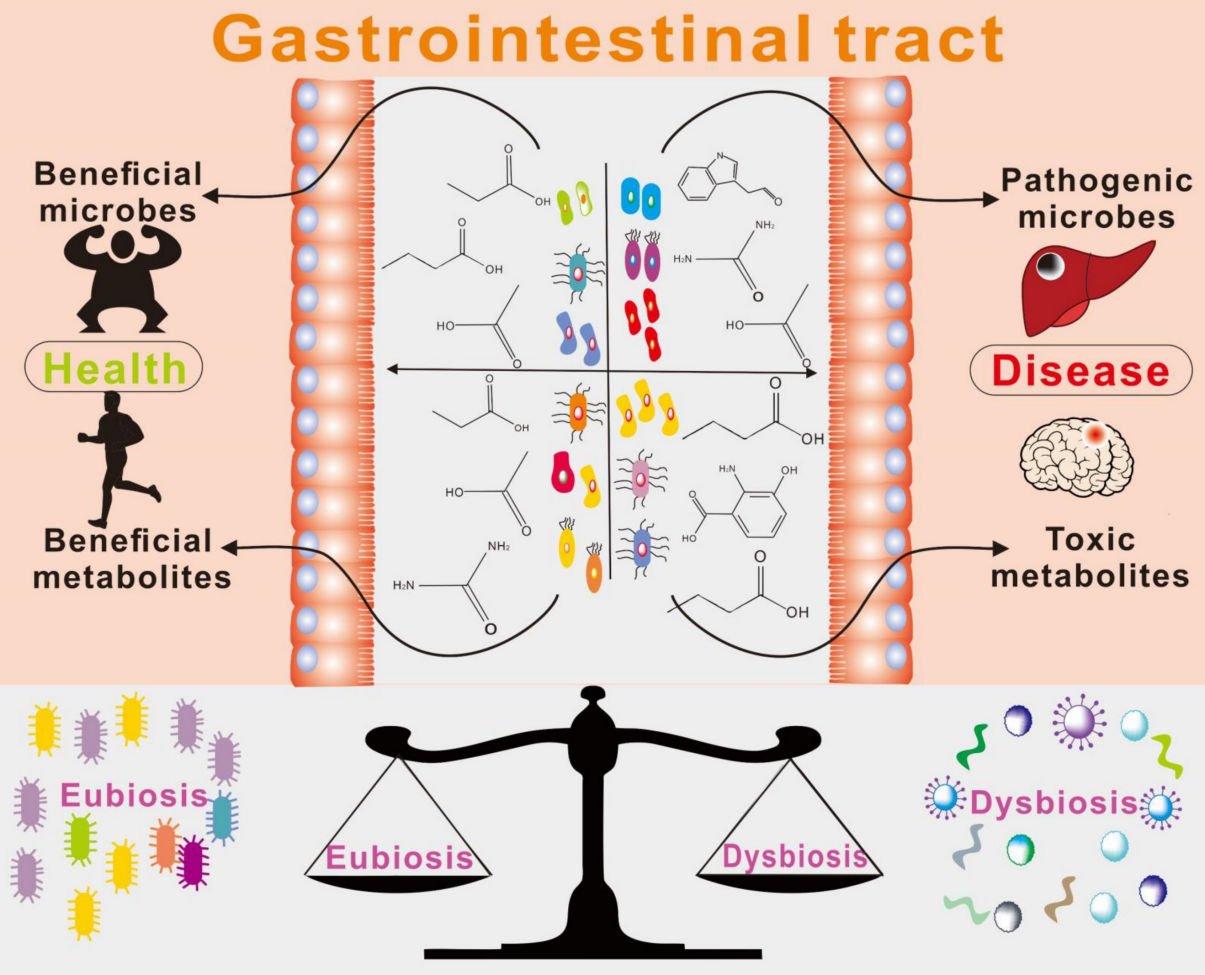
Under the steady-state conditions of the intestinal tract, various metabolites produced by the intestinal flora not only play an important role in the growth and reproduction of itself and other beneficial bacteria in the intestinal tract, but also have a positive impact on the host. When the intestinal ecology is disordered, a vicious circle will be formed between the intestinal flora and its metabolites and the host, affecting the disease state of the body.

Gut microbes play a critical role in maintaining the overall human health, including the health of the brain. Recent research has demonstrated that these microbes produce a range of metabolites that can impact brain function and may have therapeutic potential in treating brain diseases (see Figure 8). One promising approach is to use these gut microbe-mediated metabolites as a novel therapeutic strategy for brain diseases. These metabolites show great potential in the treatment of various brain diseases, including brain tumors, neuroinflammation, depression, and brain immunotherapy. Further details on this topic are described below.

### Anti-brain tumor

In recent years, there has been a growing interest in the potential therapeutic benefits of gut microbe-mediated metabolites for various diseases, including cancer. Specifically, there has been intensive research on the role of these metabolites in brain tumors. It has been demonstrated that gut microbes can produce metabolites that are capable of crossing the blood-brain barrier and directly influencing the growth and progression of brain tumors. This essay aims to explore the current state of research on the use of gut microbe-mediated metabolites for brain tumor applications 151.

Among the metabolites produced by gut microbes for brain tumor applications, butyrate stands out as one of the most promising. Butyrate is a short-chain fatty acid that is produced by gut bacteria during the fermentation of dietary fiber. Studies have shown that it has anti-tumor effects in various types of cancer, including brain tumors. Butyrate has been found to induce apoptosis (programmed cell death) in brain tumor cells while leaving healthy cells unaffected, making it an appealing candidate for use in brain tumor therapy 152,153.

Another gut microbe-produced metabolite that has shown promise in brain tumor applications is indole-3-acetate (IAA). IAA is produced by several types of gut bacteria and has been demonstrated to inhibit the growth and proliferation of brain tumor cells. IAA achieves this by blocking the activity of a protein that is necessary for the survival and growth of cancer cells. Like butyrate, IAA can selectively target cancer cells while leaving healthy cells unaffected 154.

Gut microbes-mediated metabolites have the potential to be a valuable tool in the treatment of brain tumors. The ability of these metabolites to selectively target cancer cells while sparing healthy cells makes them an attractive alternative to traditional chemotherapy and radiation therapy. Further research is necessary to fully comprehend the mechanisms by which these metabolites work, as well as their potential side effects and optimal dosages. Nonetheless, the promising results observed in preclinical studies suggest that gut microbes-mediated metabolites could play a critical role in the future of brain tumor therapy.

### Anti-neuroinflammation (Alzheimer's disease, Parkinson's disease)

Parkinson's disease and Alzheimer's disease are common and incurable diseases among the elderly in China, with no drugs available effectively solve the problem of disease progression. Therefore, more effective treatment strategies are urgently needed. In recent years, several studies have focused on the decline of the immune system and the imbalance of the gut microbiota in the elderly, and the role of the gut microbiota in age-related neurodegenerative diseases has received increasing attention 155.

Vogt and his team compared the gut microbes 25 patients with Alzheimer's disease and 25 healthy individuals and found that the diversity of gut microbes in Alzheimer's patients was reduced, with a decrease in the number of general Firmicutes and an increase in the number of Proteobacteria and Bacteroidetes.^156^. Neuroinflammation is one of the important factors in the progression of Alzheimer's disease, and the introduction of probiotics to reduce neuroinflammation through the gut-brain axis has attracted interest of researchers 157. Probiotic treatment also has shown potential efficacy in improving the cognition of Alzheimer's patients. In animal study, Bifidobacterium breve A1 administration significantly improved cognitive dysfunction in Alzheimer's disease model mice, which was extended to a clinical study 158. Elderly Alzheimer's patients were also given Bifidobacterium breve A1, and after 12 weeks of continuous treatment, the cognitive ability of patients was better maintained, with the microorganism having a certain effect on mild cognitive impairment 159. In another clinical study, selenium and probiotics co-treatment for 12 weeks showed improvements in cognitive and metabolic status 160.

There is ample evidence that healthy eating habits, including high intake of plant foods, reduced intake of saturated fat, and probiotic supplementation, can delay the occurrence of neurocognitive disorders and ultimately reduce the risk of Alzheimer's disease. Fermented milk products containing probiotics can regulate brain function and have a positive effect on cognitive function and metabolic status in Alzheimer's patients 161.

Parkinson's disease is a neurodegenerative disease often accompanied by gastrointestinal tract symptoms, and increasing evidence shows that there is a two-way connection between gut microbiota and its metabolites and Parkinson's disease. Gut microbiota contributes to the maturation and activation of microglia, and one of the pathogenesis of Parkinson's disease is microglia-induced neuroinflammation. Therefore, gut microbiota imbalance is likely to induce Parkinson's disease 162. Mouse experiments have shown that fecal microbiota transplantation (FMT) eliminated gut microbiota dysbiosis, reduced levels of SCFAs in feces, increase levels of DA and 5-HT in the striatum of Parkinson's mice, reduce activation of microglia and astrocytes in the substantia nigra and reduce levels of inflammation in the body 163.

In 2019, a Chinese hospital performed fecal microbiota transplantation by transplanting gut microorganisms from a healthy 26-year-old donor to an elderly patient with Parkinson's disease who had constipation symptoms. The treatment resulted in a significant improvement in the patient's lower limb tremor and constipation symptoms. This marks the first time that fecal microbiota transplantation has been used as a biological therapy for Parkinson's disease. However, the improvement in the patient's lower limb tremor was observed for only one week 164. Another clinical study involving 15 patients with Parkinson's disease showed that fecal microbiota transplantation was more effective when performed via colonoscopy, resulting in significant improvements in anxiety, depression, and sleep quality scores 165. Increasing clinical trials are investigating the use of probiotics in the treatment of Parkinson's disease, with more evidence showing that probiotic supplementation can improve symptoms, particularly constipation, in patients with Parkinson's disease, providing a basis for future clinical treatments 166.

### Epilepsy

Epilepsy is a neurological disorder characterized by abnormal discharge of neurons leading to increased excitability. Clinical reports have shown that there is a relationship between stress and epilepsy. Self-reported stress in patients with epilepsy triggers or worsens seizures. Patients with epilepsy have higher levels of stress hormones compared to healthy individuals, and higher levels after seizures, indicating a two-way relationship between stress and disease. Gut microbiota can contribute to the occurrence of epilepsy by producing neurotransmitters that regulate neural networks and by activating the immune system to mediate the pro-excitation of peripheral inflammation 167. In 2017, He and his team successfully treated Crohn's disease patients with a history of epilepsy using fecal microbiota transplantation. They found that epilepsy drugs were discontinued after the first FMT, and no symptoms of epilepsy recurrence were observed during the follow-up of 20 months. This is the first report of successful treatment of epilepsy using FMT 168.

Of course, the role of probiotics in the clinical treatment of epilepsy cannot be ignored. Gómez-Eguílaz and his team administered mixed probiotics treatment to patients with resistant epilepsy. During the four-month treatment, the number of seizures and quality of life of patients were evaluated. It was found that the number of seizures in some patients was greatly reduced, and their quality of life was also significantly improved, proving that probiotics treatment may be used as a complementary therapy for epilepsy treatment 169. Samaneh and his colleagues used a probiotic mixture (Lactobacillus rhamnose, Lactobacillus reuteri, and Bifidobacterium infantis) to treat PTZ-induced epileptic rats. The results showed that compared to the control group, the frequency and severity of seizures in the experimental group were significantly reduced, and the level of inhibitory neurotransmitter GABA was increased 170.

### Immunity therapy

**Figure 9:**
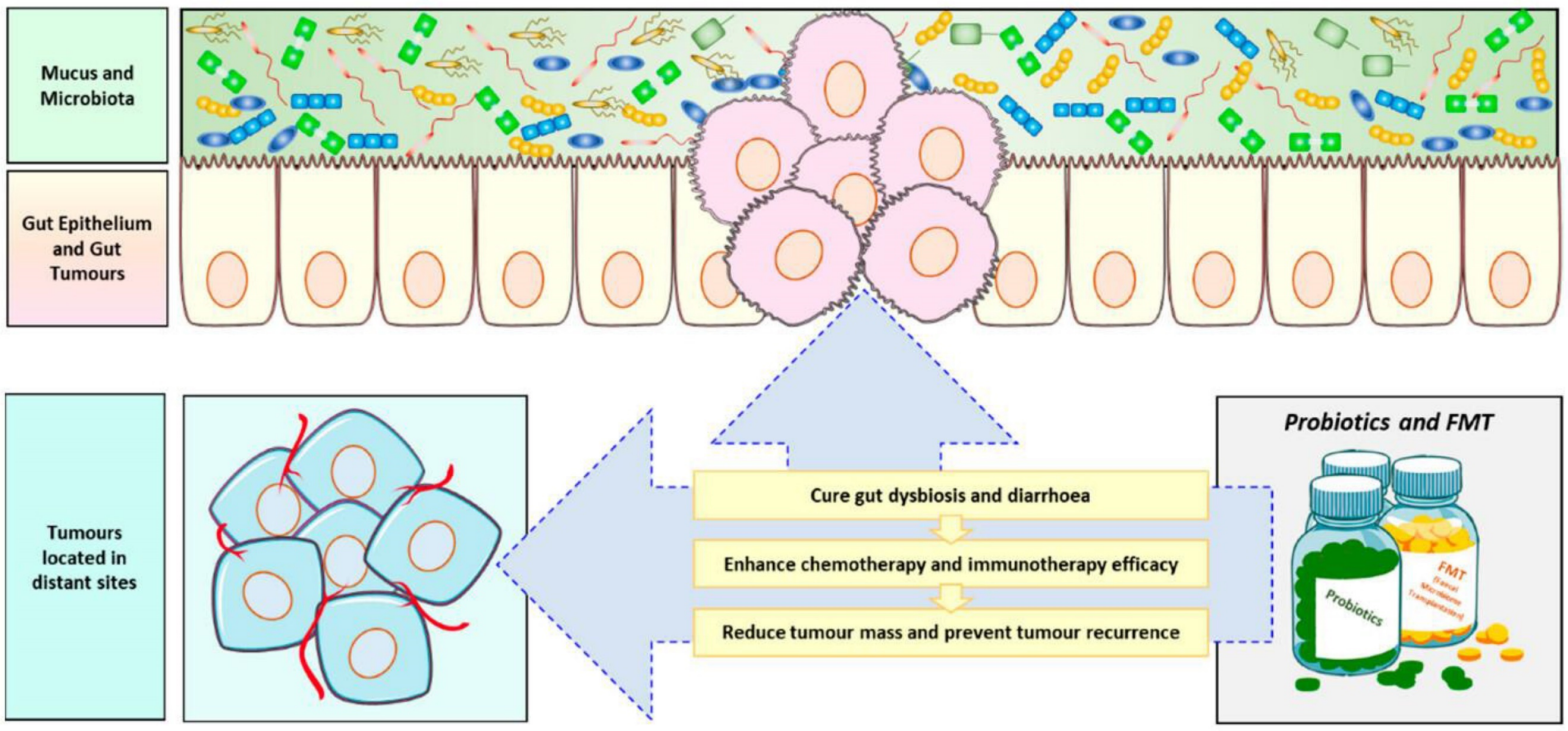
Role of probiotics in anticancer therapy^174^. Probiotics and fecal microbiota transplantation (FMT) is now often used as an adjuvant anti-cancer therapy to combat the body's ecological imbalances caused by cancer chemotherapy and radiotherapy, and to improve treatment outcomes, especially immunotherapy, while preventing tumor recurrence.

The mammalian gastrointestinal tract is a complex ecosystem, consisting of numerous gut microbiota and the host immune system, which complement each other. Specific microbiota in the gut can promote the maturation of the host immune system. Mazmanian and his team studied the effect of colonizing Bifidobacterium fragile in the gut of sterile mice on the immune system of mice. The results showed that CD_4_ T cells in the spleen of mice increased, and almost completely recovered to normal levels 171. Studies have shown that the abundance of gut microorganisms can affect the efficacy of tumor chemotherapy. Therefore, using probiotics and fecal microbiota transplantation as adjuvant therapies can help increase the effectiveness of cancer treatment. (Figure 9) The use of cyclophosphamide in sterile mice or mice treated with antibiotics to treat tumors is not ideal for the treatment of cyclophosphamide resistance. This may be due to the fact that the effect of cyclophosphamide in the treating tumors depends, to some extent, on its ability to stimulate anti-tumor immune response, which in turn depends on the translocation of specific gut microbiota to stimulate the production of immune response. A reduction in immune response is observed in sterile mice 172. In another study, it was found that the use of specific bacteria such as Bifidobacterium fragile can overcome the resistance of sterile mice to CTLA-4 immunotherapy 173.

Cancer has always been a major challenge for humanity to overcome. Current treatment methods cannot cure most cancers, and a key feature of cancer is its immune function deficiency. Cancer cells can create an immunosuppressive microenvironment, allowing them to grow and escape immune destruction. In recent years, new evidence has shown that immunotherapy can offer hope in the treatment of cancer, particularly immune checkpoint blockade, which reactivates the immune function of immune cells by blocking immune checkpoints to restore their anti-tumor activity. Gut microorganisms can not only regulate the body's own immune function, but also work with immune checkpoint inhibitors to reduce adverse reactions 175.

For example, Marie's study on the use of antibodies against CTLA-4, a major regulator of T-cell activation, for cancer immunotherapy found that its anti-tumor effect may depend on the different types of Bacteroides in the body 173. Additionally, the gut microbiome can also enhance the effectiveness of cancer immunotherapy. For instance, when mice with melanoma were orally administered Bifidobacterium alone, the degree of tumor inhibition was consistent with the use of a programmed cell death protein ligand 1(PD-L1) specific antibody alone, while the combined use almost eliminated tumor growth. The mechanism may be that the function of dendritic cells is enhanced, resulting in improved initiation and accumulation of CD8 T cells in the tumor microenvironment 176. Fecal microbiota transplantation can also overcome PD-1 resistance in patients with advanced melanoma 177. Furthermore, through the identification of patients with early progression and long-term response to second-line treatment of non-small cell lung cancer with nivolumab, the data initially showed that SCFAs lysine and nicotinic acid were significantly associated with long-term treatment efficacy 178. Studies have demonstrated that gut microorganisms and their metabolites are also promote the production of regulatory T cells. SCFAs can promote the differentiation of regulatory T cells 179 and increase the size of regulatory T cell pools 180.

Furthermore, the presence of gut microorganisms can help mitigate complications during cancer immunotherapy. For example, Lactobacillus reuteri has been shown to completely eliminate colitis induced by immune checkpoint inhibitors 181. However, not all gut microbes are beneficial for treatment. Oral administration of neomycin significantly reduces the number of Gram-positive bacteria in the gut, leading to a decrease in the body's immunity to respiratory viruses 182. Prebiotic treatment can modify the neuroimmune response through SCFAs, reducing LPS-induced anxiety in mice 183. Although adoptive cell therapy (ACT) is a promising strategy of cancer immunotherapy cancer, it has limitations, especially in brain tumors due to the lack of specific antigens and heterogeneity of surface cell antigens. Even when effective, there are significant adverse reactions.

### Other

Studies have shown that FMT of young donor microbiota into elderly mice can improve age-related central nervous system inflammation and slow down the loss of key functional proteins in the eyes 184. Hepatic encephalopathy is a common condition in patients with liver injury, and its clinical treatment is often revolving around the gut. The commonly used drugs are mainly prebiotic lactulose and antibiotic rifaximin. The treatment mechanism involves changing the composition of gut microorganisms, leading to a decrease in gut ammonia production. Probiotics can also alter the composition of the gut microbiome and can be used as a second-line treatment for hepatic encephalopathy. The recurrence rate of patients treated with probiotics is much lower than that of the untreated group 185.

Studies have shown that supplementation of prebiotics can reduce the body's stress immune response, improve symptoms such as depression and anxiety, increase the expression of brain-derived neurotrophic factor, and improve cognitive status. Additionally, prebiotics supplementation can increase the level of SCFAs in the body, improve the social behavior and sleep pattern of autistic patients, and reduce the anxiety score of patients with irritable bowel syndrome 48. Maria and her team evaluated the effects of Bifidobacterium longum on anxiety and depression in patients with irritable bowel syndrome by treating 22 patients with irritable bowel syndrome and diarrhea with Bifidobacterium longum for six weeks. The results showed that Bifidobacterium longum as a probiotic reduced the incidence of depression in patients with irritable bowel syndrome and improved their quality of life but did not reduce the patient's anxiety score. The reason may be that the brain's limbic system is less responsive to negative emotions 186. Kato-Kataoka and his team gave healthy students who were about to take the test 8 weeks of milk fermented by Lactobacillus casei or placebo milk. Before the test, it was found that the cortisol level in the saliva of the experimental group was significantly reduced, which alleviated the gastrointestinal tract symptoms caused by stress 187.

Propionic acid, a gut metabolite of Clostridium difficile, has been linked to autism due to increased levels found in the gut. Researchers have induced autism in rats using propionic acid and used probiotics (Bifidobacterium and Lactobacillus) to treat them. Afaf El-Ansary and colleagues found that probiotic treatment reduced glutamate excitotoxicity by increasing GABA and Mg2+ consumption and decreasing glutamate levels *in vivo* 188. A 19-week clinical trial of probiotics (mainly lactobacilli and bifidobacteria) for children with autism, anxiety and gastrointestinal tract symptoms showed that the gastrointestinal tract symptoms of the experimental group were significantly improved compared to the placebo 189. Kang and his team transplanted healthy gut microbiota into autistic children and observed improvements in symptoms in 18 patients, as well as a beneficial change in the gut environment. The improvement continued for 8 weeks after the end of treatment, indicating long-term therapeutic benefits of FMT 190. Additionally, depressed mice exhibited decreased levels of lactobacilli in the gut tract, while supplementation of probiotics significantly increased the abundance of lactobacilli and improved anxiety and depression-like symptoms induced by chronic mild stress in mice 191.

There is a significant relationship between gut microbiota and multiple sclerosis (MS). Borody and his team observed the conditions of three patients with multiple sclerosis with different complications after treatment with FMT. The patients' gut and neurological symptoms were improved, and they speculated that FMT eliminated occult gut pathogens that caused MS 192.

**Figure 10:**
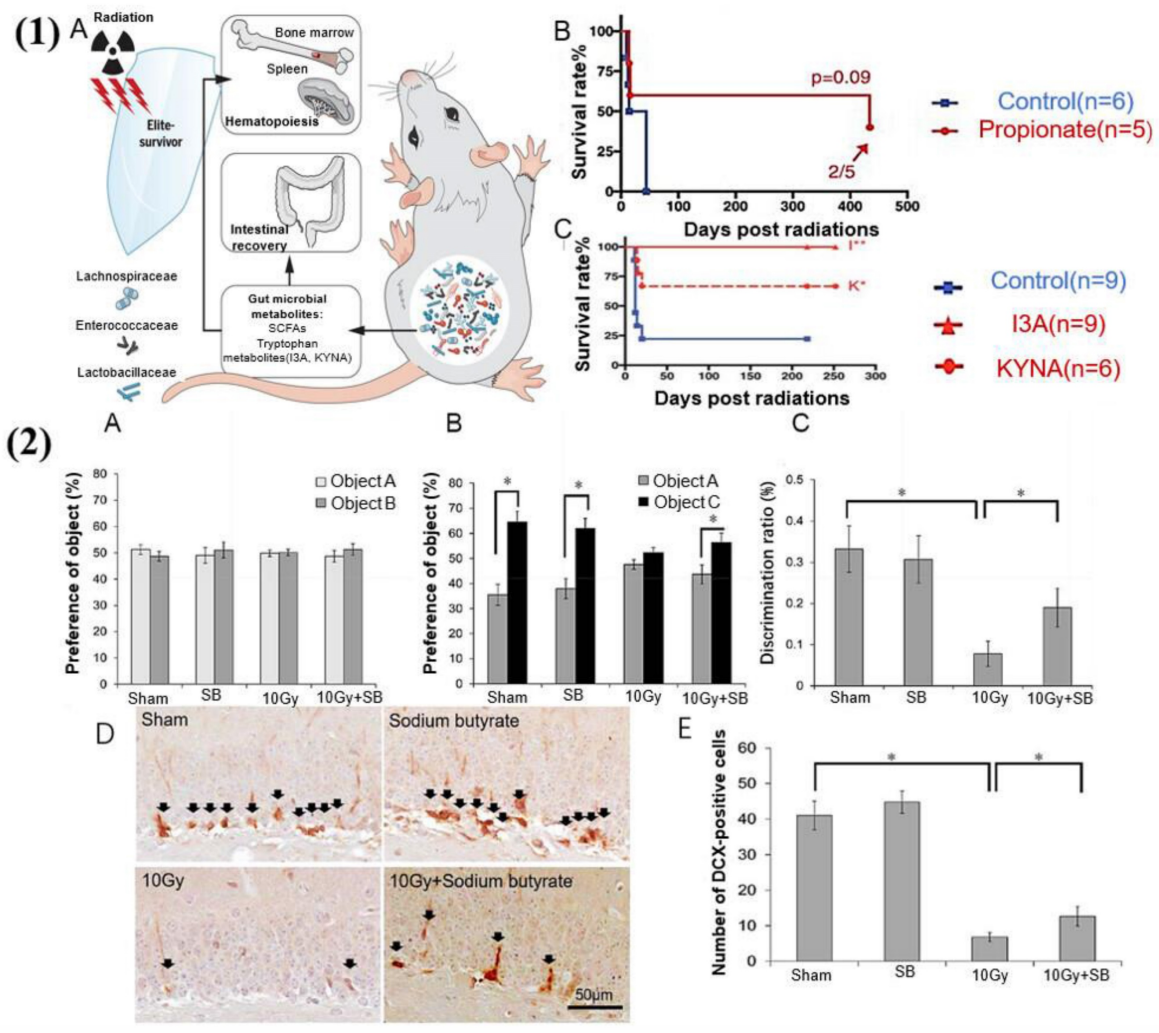
The gut microbiota and its metabolites have a protective effect against radiation-induced damage. (1) Gut microbiota and metabolites mediate radioprotection 194. (A) Gut microbes and certain metabolites modulate host resistance to high doses of radiation by promoting hematopoiesis and gastrointestinal recovery. (B) SCFAs suppress the radiation-induced death and damage. Significantly reduce mortality in irradiated mice. (C) Untargeted metabolomics reveals tryptophan metabolites as potent radioprotectants. Significantly prolongs the survival of irradiated mice. (2) Sodium butyrate prevents radiation-induced cognitive impairment 196. (A) There was no difference in the preference for objects between the groups of mice during the object recognition test phase. (B) During the test phase, the vehicle-treated irradiation group (10 Gy group) exhibited reduced preference for a novel object (cylinder shape (object C)) compared to the vehicle-treated sham group (sham group), and injection of sodium butyrate attenuated the radiation-induced cognitive impairment. (C) The discrimination ratio was markedly lower in the 10 Gy group, and SB treatment inhibited the decrease of discrimination ratio by radiation exposure after 30 days. (D) DCX (doublecortin) immunoreactivity (arrows) in the DG (dentate gyrus) of the hippocampus. (F) Quantification of DCX-immunoreactive cells. SB attenuated radiation-induced decrease in neurogenesis.

It is well known that the most common adverse symptom of radiation therapy for cancer is gastrointestinal dysfunction, which can cause further damage to the body and terminate treatment 193. However, gut microbial metabolites appear to be inextricably linked to radiation protection. It has been reported that SCFAs and tryptophan metabolites can reduce proinflammatory cytokines such as tumor necrosis factor-α, interleukin-6, and interferon-γ and promote the anti-inflammatory cytokines, which are vital mediators of radiation-induced damage 194 (see Figure 10). Moreover, FMT can also play a therapeutic role in radiation-induced diseases, which is of great significance for military and public health issues. It can especially improve the prognosis of cancer after radiotherapy 195.

However, when it comes to treating diseases mediated by these metabolites, the process can be slightly more complex, as the efficacy of drug treatment is influenced by the gastrointestinal tract and the blood-brain barrier. The blood-brain barrier is the central nervous system's most important protective mechanism, allowing certain small molecules to enter the brain while preventing harmful substances from entering. However, it also limits drug delivery and increases the difficulty of treating brain diseases 197. Recently, nanoparticle-mediated drug delivery has emerged as an effective way to treat brain diseases 198. It can not only improve drug targeting to the brain but also improve the bioavailability of drugs 199, simplify the treatment process, and improve patient compliance. We have concluded that central nervous system diseases are often related to gut and its metabolites. Clinically, most of them are treated by directly supplementing or reducing related gut and metabolites, and researchers have increasingly become interested in exploring the combination of nanomaterials with these treatments.

For instance, Liu and her colleagues developed a method for delivering mesoporous silica nanospheres loaded with Bifidobacteria to the intestine through nasal administration. This approach overcomes the harsh acidic and bile salt conditions in the gastrointestinal tract that can destroy the beneficial bacteria. The study confirmed that this approach can inhibit intestinal inflammation, reduce brain Aβ load, and improve symptoms in patients with Alzheimer's disease (AD) 200. Plant polyphenols can be metabolized into small molecule phenolic compounds in the gut, and have been shown to be non-toxic treatments for central nervous system diseases. Incorporating these polyphenols into nanomaterials based on polyethylene glycol, polylactic acid, etc., can enhance their permeability through the blood-brain barrier, providing an effective way to treat AD 201,202. In addition, Qiao and her colleagues evaluated the protective effect of selenium-rich nanoparticle Lactobacillus casei ATCC 393 on chemically induced AD model mice and found that it has a potential therapeutic effect on cognitive impairment compared to Lactobacillus casei alone 203.

## Conclusions and Future Directions

Gut microbes-mediated metabolites have emerged as a potential new frontier in the treatment of brain diseases. The gut-brain axis, a complex communication network between the gut and the brain, has been found to play a crucial role in the regulation of brain function and the development of brain diseases. This has led to a growing interest in the therapeutic potential of gut microbes-mediated metabolites in the treatment of brain diseases.

Gut microbes-mediated metabolites have shown promising results in the treatment of neurological diseases such as Parkinson's disease and Alzheimer's disease. For example, butyrate, a short-chain fatty acid produced by gut bacteria, has been shown to improve motor function and reduce neuroinflammation in animal models of Parkinson's disease. Similarly, the metabolite indole-3-propionic acid (IPA) has been found to protect against neuronal damage in Alzheimer's disease. In addition to neurological diseases, gut microbes-mediated metabolites have also shown potential in the treatment of psychiatric disorders such as depression and anxiety. Studies have found that certain gut microbes can produce metabolites that act on the central nervous system and regulate mood and behavior. For example, the metabolite kynurenine has been found to play a role in the development of depression, while butyrate has been shown to improve anxiety-like behavior in animal models.

While the potential of gut microbes-mediated metabolites in the treatment of brain diseases is promising, there are still many challenges to be addressed. One of the major challenges is understanding the complex interactions between gut microbes, their metabolites, and the brain. Another challenge is developing effective delivery methods for these metabolites, as they must be able to cross the blood-brain barrier to exert their therapeutic effects. However, recent advancements in neuroscience and microbiome research have provided new opportunities for developing targeted and effective treatments for brain diseases.

In summary, the microbial metabolism substance of the gut, which serves as the primary material foundation of the gut-brain axis, offers a path forward for understanding brain illnesses formation and developing treatments for them. The future of gut microbes-mediated metabolites in the treatment of brain diseases is bright. The potential of these metabolites to regulate brain function and ameliorate the symptoms of neurological and psychiatric disorders is a promising area of research. Continuing effort and advances in the fields of materials science and engineering, chemistry, biology, and computer science are expected to lead to success in precise manipulation of the human gut microbiota-derived metabolites and translating into clinical applications in the near future.

## Figures and Tables

**Table 1:** Gut metabolites and related microbiota and their associated diseases.

Gut metabolites	Structure	Gut microbiota	Disease	Reference
TMAO	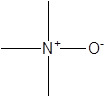	Prevotella	Atherosclerosis Heart failure Hypertension Alzheimer's disease Osteoporosis	37 42 44 77
Urea	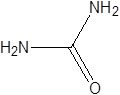	Colibacillus	Renal failure	78
SCFAs (Acetic acid, Propionic acid, Butyric acid)	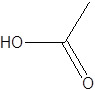 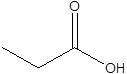 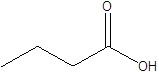	Acetobacteraceae Lachnospiraceae Ruminococcaceae Erysilotrichaceae Roseburia Bacteroides Faecalibacterium Blautia Anaerostipes Odoribacter Akkermansia	Autism spectrum disorder Parkinson's disease Multiple sclerosis (MS) Depression Ischemic stroke Amyotrophic lateral sclerosis Colon cancer	19 32 74 79 80 81 82 83
Vitamin D	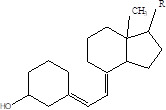	Salmonella typhimurium	Rickets Osteoporosis	84
Vitamin B6	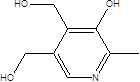	Actinobacteria Bacteroidetes Proteobacteria phyla	Pellagra	60 85
Galic acid (GA)	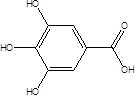	Lactobacillus plantarum Bacillus subtilis	High blood pressure Colon cancer	86
Equol	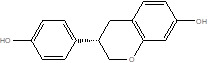	Adlercreutzia equolifaciens Lactococcus garvieae Slackia equolifaciens	Atherosclerosis	87
Indole-3-aldehyde (IAld)	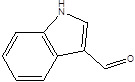	Clostridium Peptostreptococcus russellii Lactobacillus spp Lactobacillus reuteri	Colitis	88 89 90 91
Indole-3-acetic-acid (IAA)	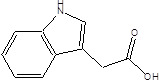	Bacteroides thetaiotaomicron Bacteroides eggerthii Bifidobacterium adolescentis Bifidobacterium spp. Bifidobacterium pseudolongum Clostridium sporogenes Clostridium difficile Clostridium putrefaciens Clostridium sticklandii Clostridium subterminale Escherichia coli Eubacterium hallii Eubacterium cylindroides Parabacteroides distasonis	Ankylosing spondylitis	90 92 93 94
Indole-3-lactic acid	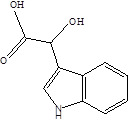	Anaerostipes hadrus Anaerostipes caccae Bacteroides eggerthii Bacteroides ovatus Bacteroides fragilis Clostridium perfringens Clostridium sporogenes Clostridia Escherichia. coli Eubacterium rectale Eubacterium cylindroides Lactobacillus murinus Lactobacillus paracasei Lactobacillus reuteri Megamonas hypermegale Parabacteroides distasonis	Necrotizing colitis	91 95 96 97 98
Indole-3-propionic acid (IPA)	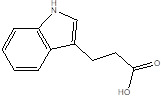	Bacteroides Clostridium sporogenes Clostridia Peptostreptococcus spp. Escherichia. coli Lactobacillus Peptostreptococcus russellii Peptostreptococcus anaerobius Peptostreptococcus stomatis	Metabolic syndrome Liver fibrosis Alzheimer's disease Steatohepatitis	89 90 93 95 99 100
Indole-3-acetaldehyde (IAAld),	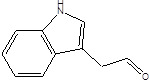	Escherichia coli	Inflammatory bowel disease Obesity Metabolic syndrome	101 102
3-hydroxyanthranilic acid (3-HAA)	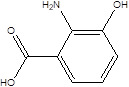	Pseudomonas fluorescens strain KU-7 Burkholderia cepacia J2315	Crohn's disease Ulcerative colitis	103 104 105
Indoleacrylic acid	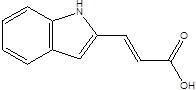	Clostridium sporogenes Peptostreptococcus spp. Peptostreptococcus. russellii Peptostreptococcus anaerobius Peptostreptococcus stomatis	Inflammation Food allergies	89 93 106 107
Tryptamine	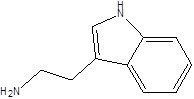	Bacteroides Clostridium sporogenes Escherichia. coli Firmicutes C. sporogenes Ruminococcus gnavus	Epilepsy Anxiety Depression Irritable bowel syndrome Osteoporosis	108 109 110 111
Catecholamine	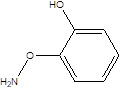	Candidatus arthromitus Clostridiaceae Lactobacillus Pseudomonadaceae	Depression Liver fibrosis Fatty liver diseases Liver cancers	112
GABA	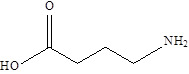	Lactobacillus brevis Bifidobacterium dentium	Epilepsy	113
Dopamine	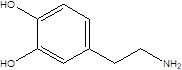	Escherichia coli Klebsiella pneumoniae Pseudomonas aeruginosa Shigella sonnei Staphylococcus aureus	Parkinson's disease	114 115
Bile Acids	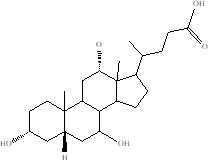	Lachnospira Clostridium Enterobacteriaceae, Streptococcus, Fusobacteria, Gemella Rothia	Irritable Bowel Syndrome	116
Hypoxanthine	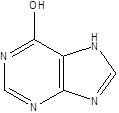	Pseudomonadaceae	Depression Atherosclerosis	117 118
3-hydroxypristanic	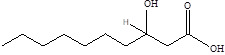	Clostridiaceae Candidatus Arthromitus	Depression	117
L-Carnitine	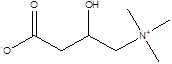	Lactobacillus	Depression Heart disease	117 119
Threonic acid	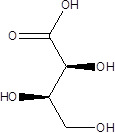	Clostridiaceae Candidatus Arthromitus	Depression	117
